# 再生障碍性贫血小鼠模型中免疫活化T细胞动力学研究

**DOI:** 10.3760/cma.j.issn.0253-2727.2022.07.009

**Published:** 2022-07

**Authors:** 伟望 李, 若难 李, 乐乐 张, 秋怡 马, 浩渊 李, 文君 汪, 进 毛, 雅婧 初, 卫平 袁, 均 施

**Affiliations:** 中国医学科学院、北京协和医学院血液病医院（中国医学科学院血液学研究所），实验血液学国家重点实验室，国家血液系统疾病临床医学研究中心，细胞生态海河实验室，天津 300020 State Key Laboratory of Experimental Hematology, National Clinical Research Center for Blood Diseases, Haihe Laboratory of Cell Ecosystem, Institute of Hematology & Blood Diseases Hospital, Chinese Academy of Medical Sciences & Peking Union Medical College, Tianjin 300020, China

**Keywords:** 贫血，再生障碍性, 小鼠模型, T细胞, 动力学, 自身免疫, Anemia, aplastic, Mouse model, T cells, Kinetics, Autoimmunity

## Abstract

**目的:**

探索再生障碍性贫血（AA）小鼠模型中供者来源T细胞不同时间点动力学变化。

**方法:**

构建AA小鼠模型，分别于不同时间点采用流式细胞术测定模型小鼠脾脏与骨髓内供者T细胞比例、活化分子表达、细胞周期及功能亚群，评估不同时期T细胞功能状态。

**结果:**

①半致死剂量照射联合主要组织相容性抗原（MHC）半相合的淋巴结细胞输注成功构建T细胞免疫介导的AA小鼠模型。②AA小鼠脾脏中供者T细胞从移植第3天后开始明显浸润，并逐渐出现CD4^+^/CD8^+^比例倒置，第5天开始进入骨髓，以CD8^+^细胞浸润为主。③CD69在供者CD4^+^细胞中表达高峰晚于CD8^+^细胞，CD25在CD4^+^细胞与CD8^+^细胞中表达水平的变化趋势相同，但在CD8^+^细胞中的表达高于CD4^+^细胞。④脾脏内供者CD4^+^细胞S/G_2_/M期比例在移植后第3天即达高峰，约12％，而CD8^+^细胞中S/G_2_/M期比例在移植后第5天达高峰，约20％，且两者在进入骨髓后S/G_2_/M期比例均再次升高，但移植第3天后其比例在脾脏与骨髓CD8^+^细胞中持续高于CD4^+^细胞。⑤脾脏内免疫活化的T细胞经历短暂的中枢记忆T细胞（T_CM_）阶段后迅速分化为效应记忆T细胞（T_EM_），进入骨髓后部分T_EM_分化为效应细胞进一步发挥效应功能。

**结论:**

AA小鼠模型中供者T细胞进入异体后迅速活化，5天内达增殖高峰，并完成向T_EM_细胞的分化，5天后开始进入骨髓进一步增殖损伤造血。

## Abstract (en)

**Objective:**

To explore the dynamic changes of donor derived T cells at different time points in the aplastic anemia mouse model.

**Methods:**

The aplastic anemia mouse model was induced and then the proportion of infiltrated donor derived T cells in spleen and bone marrow, expression of activation molecular markers, cell cycle and functional subsets were measured by flow cytometry at different time points to evaluate the functional status of T cells in different periods.

**Results:**

①T cell immune-mediated aplastic anemia mouse model was successfully established by half lethal dose irradiation combined with major histocompatibility antigen (MHC) haploidentical lymph node cells infusion. ②The donor derived T cells began to infiltrate significantly in the spleen of aplastic anemia mouse from the 3rd day after transplantation and the ratio of CD4^+^/CD8^+^ gradually inverted. After the 5th day, they gradually entered the bone marrow, predominated by CD8^+^ cells. ③The expression peak of CD69 in donor CD4^+^ cells was later than that in CD8^+^ cells. The trend of CD25 expression in CD4^+^ cells was the same as that in CD8^+^ cells, but the expression level in CD8^+^ cells was higher than CD4^+^ cells. ④The proportion of donor CD4^+^ cells in S/G_2_/M phase reached the peak in spleen, about 12%, within 3 days after transplantation, while a higher level in CD8^+^ cells, which was about 20%. And the proportion of both CD4^+^ and CD8^+^ cells in S/G_2_/M phase increased again after entering bone marrow, which was continued to be higher in CD8^+^ cells than that in CD4^+^ cells after 3 days of transplantation. ⑤Immune activated T cells in the spleen rapidly differentiated into effector memory T cells (T_EM_) after a short central memory T cell (T_CM_) stage. After entering the bone marrow, some T_EM_ differentiated into effector cells to further function.

**Conclusion:**

In the aplastic anemia mouse model, donor derived T cells activated rapidly after entering the allogenic recipient, reached its proliferation booming period and differentiated into T_EM_ cells within 5 days. After 5 days, they began to enter the bone marrow to continue proliferate and damage hematopoiesis.

再生障碍性贫血（AA）是最常见的获得性骨髓造血衰竭性疾病，自身T细胞免疫活化后介导的骨髓造血损伤是AA发生的核心病理机制[1]–[2]。免疫介导的AA小鼠是研究AA发病机制与治疗靶点的常用模型，其基本原理是主要组织相容性抗原（MHC）半相合的供者T细胞在受体小鼠体内免疫活化后损伤受体小鼠骨髓造血。AA小鼠模型疾病程度与生存时间因建立模型的实验条件不同而有所不同[3]–[5]，常规模型小鼠在移植后2～3周内死亡，生存时间较短，提示在此过程中供者T细胞功能状态变化迅速。因此，我们对AA小鼠模型中不同时间点T细胞动力学进行了研究，精准把握AA小鼠模型发病过程中T细胞发挥作用的关键时间点，为充分利用此模型进行进一步研究提供更多依据。

## 材料与方法

一、主要试剂与仪器

流式抗体CD45.1-APC、CD45.1-PerCP-Cy5.5、CD3-PE、CD4-PE-Cy7、CD8-APC、CD8-APC-Cy7、CD25-APC-Cy7、CD69-FITC、CD44-FITC、Ki-67-FITC均购于美国Biolegend公司，CD62L-BV421购于美国BD公司。细胞周期检测试剂盒IntraSure Kit购于美国BD公司，红细胞裂解液购于北京索莱宝科技有限公司，Hochest33342、10％甲醛固定液购于美国Merck公司，恩诺沙星溶液购于德国拜耳公司。流式细胞仪Canto Ⅱ与LSR Ⅱ购于美国BD公司，全自动血液体液分析仪SYSMEX XN-1000购于日本希森美康公司，X线辐照仪购于美国Rad Source公司。

二、实验动物

健康B6.SJL-Ly5.1（CD45.1，以下简称B6）小鼠购于杰克森实验室，健康CB6F1小鼠购于北京维通利华实验动物技术有限公司，所用小鼠均为雌性，8～12周龄，体重（20±2）g。小鼠均饲养于SPF环境，所有动物实验操作均符合动物伦理委员会要求。

三、AA小鼠模型的构建

无菌条件下摘取B6小鼠腹股沟、腋窝、颈部等部位淋巴结，研磨后经300目尼龙膜过滤制作淋巴结单细胞悬液。CB6F1小鼠经X射线照射4 Gy，照射前一天给予含2‰恩诺沙星的饮用水预防感染，照射后随机分为两组，分别于照射后4～6 h 给予AA组小鼠尾静脉注射5×10^6^ B6小鼠淋巴结细胞，TBI组小鼠注射同等体积的PBS。同时留取部分B6小鼠淋巴结细胞作为d 0样本进行流式检测。

四、AA小鼠模型检测

造模后第14天取6只AA组与4只TBI组CB6F1受鼠尾静脉血进行血细胞计数检测，并分别取双侧髂骨、股骨、胫骨，冲出骨髓细胞，经红细胞裂解液处理后计数并行进一步流式检测，同时取胸骨放入10％甲醛固定液中固定24 h后行病理检测，综合评估骨髓造血状况。移植后第3、5、7、9天分别取3～4只受鼠脾脏与骨髓细胞进行流式检测，分析供者T细胞动力学变化。

五、流式细胞术检测

脾细胞与骨髓细胞分别4 °C 350×*g*离心5 min，加入1 ml红细胞裂解液室温处理10 min，再离心后各加入1 ml PBS重悬细胞沉淀，计数后取相应细胞量在4 °C标记CD45.1、CD3、CD4、CD8等表面分子抗体30 min，然后进行流式细胞术检测。检测细胞周期样本根据IntraSure Kit说明书进行处理，标记表面分子抗体后Reagent A室温处理5 min，Reagent B室温处理30 min，同时标记Ki-67抗体，流式细胞仪检测前加入Hochest33342。

六、统计学处理

统计学分析采用GraphPad Prism8.0软件，所有数据采用平均数±标准差表示，组间比较采用独立样本*t*检验，*P*<0.05为差异有统计学意义。

## 结果

一、MHC半相合的T细胞浸润骨髓损伤小鼠骨髓造血

AA组小鼠出现明显体重下降，逐渐呈现濒死状态；TBI组小鼠体重逐渐恢复，生存状态良好。淋巴结细胞输注后第14天检测受体小鼠骨髓衰竭程度，外周血细胞计数结果显示AA组小鼠WBC、HGB、PLT水平均显著低于TBI组，出现明显三系减低（图1A）。TBI组小鼠骨髓有核细胞计数恢复至接近正常水平，而AA组小鼠骨髓有核细胞较TBI组下降约20倍（图1B），胸骨HE染色也提示AA组小鼠骨髓明显空虚，TBI组造血基本恢复（图1C）。进一步的免疫表型分析发现TBI组小鼠照射后骨髓内T细胞比例呈低水平，而AA组小鼠骨髓中T细胞明显浸润（图1D），表明MHC半相合的T细胞浸润受体小鼠骨髓损伤造血。

**图1:**
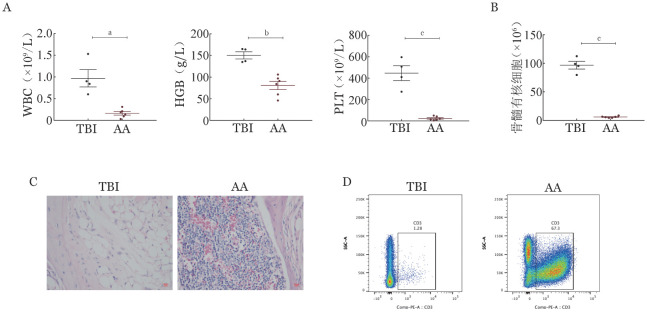
全身照射（TBI）联合5×10^6^ B6.SJL-Ly5.1小鼠淋巴结细胞输注损伤CB6F1小鼠骨髓造血（^a^*P*<0.01、^b^*P*<0.001、^c^*P*<0.0001） TBI：TBI后输注PBS对照组小鼠；AA：TBI后输注供体小鼠淋巴结细胞构建的再生障碍性贫血模型小鼠。A：血常规；B：骨髓有核细胞计数；C：胸骨HE染色；D：骨髓T细胞比例

二、AA小鼠体内供者来源T细胞浸润程度变化

经CD45.1^+^CD3^+^标记供者来源T细胞，动态监测其在AA小鼠体内浸润程度变化，发现移植后第3天AA小鼠脾脏内供者T细胞比例接近20％，T细胞比例与绝对值结果均显示第3、5天T细胞在脾脏内浸润程度迅速增加，且从第5天开始逐渐进入骨髓并继续扩增（图2A）。同时，脾脏内供者T细胞中CD4^+^/CD8^+^比值在移植后逐渐下降至比例倒置，T细胞进入骨髓后仍维持以CD8^+^细胞为主的状态（图2B）。

**图2:**
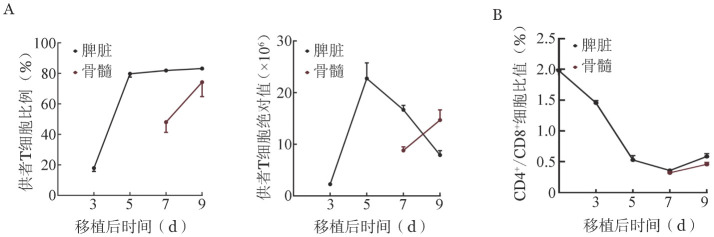
T细胞在再生障碍性贫血小鼠脾脏与骨髓内浸润程度变化 A：供者来源T细胞比例与绝对计数；B：供者来源CD4^+^/CD8^+^ T细胞比值

三、脾脏内供者来源T细胞免疫活化程度变化

通过监测CD69、CD25的表达水平来评估供者T细胞在AA模型中活化程度的变化。结果显示，输注后第3天CD69在CD8^+^细胞中的阳性细胞比例及其平均荧光强度（MFI）均高于与CD4^+^细胞，此后，其在CD4^+^细胞中的表达水平在第5天达高峰，而在CD8^+^细胞中的表达逐渐下降（图3A）。同样，CD25在CD4^+^与CD8^+^细胞中也在输注后5天内达高峰，且在CD8^+^细胞中表达几乎持续高于CD4^+^细胞（图3B）。以上结果均提示，供者T细胞在AA模型中经异体抗原刺激活化后CD8^+^细胞比CD4^+^细胞活化更早，活化程度更高。

**图3:**
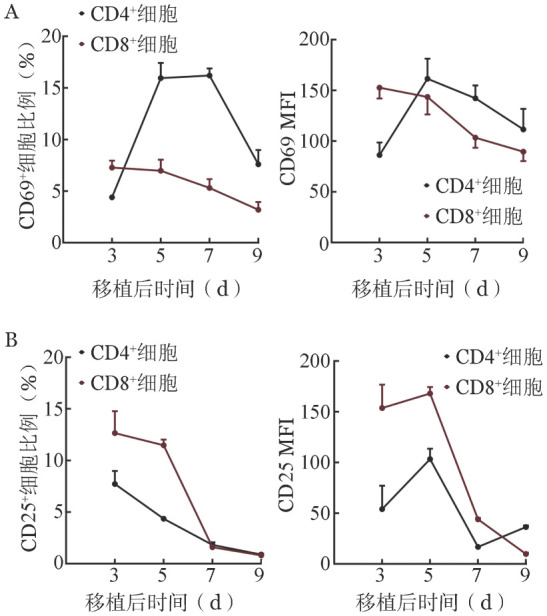
再生障碍性贫血小鼠脾脏内供者来源T细胞CD69（A）与CD25（B）免疫活化程度变化

四、体内免疫活化T细胞细胞周期变化

T细胞被免疫活化后开始增殖，为进一步发挥效应功能准备。通过细胞周期分析，我们发现AA小鼠脾脏内供者CD4^+^与CD8^+^细胞中S/G_2_/M期细胞比例在移植后第3天接近，均在12％左右。此后其在CD8^+^细胞中的比例在第5天接近20％，并且在移植后9天内均维持在10％以上，而CD4^+^细胞中S/G_2_/M期细胞比例在第3天后持续下降至不足4％（图4A）。同时，结果显示移植后第7天骨髓内S/G_2_/M期细胞比例在CD4^+^与CD8^+^细胞中比例均高于脾脏，提示T细胞进入骨髓后进一步增殖。与脾脏内T细胞相同的是，CD8^+^细胞中S/G_2_/M期细胞在移植后中第7、9天维持较高比例，而在CD4^+^细胞中比例开始下降（图4B），表明骨髓内的CD8^+^细胞增殖速度仍高于CD4^+^细胞。

**图4:**
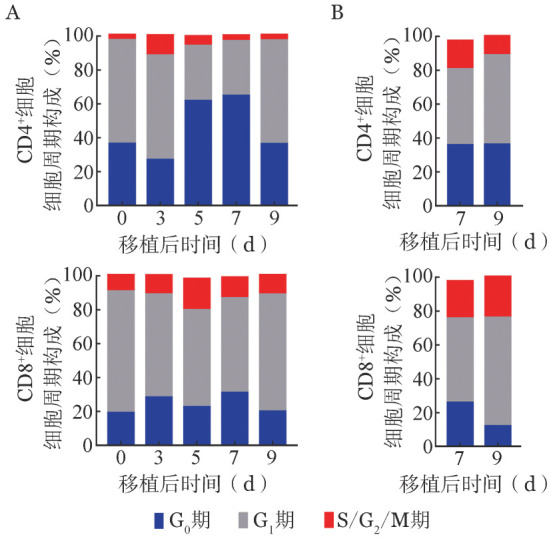
再生障碍性贫血（AA）小鼠脾脏（A）与骨髓（B）内免疫活化T细胞细胞周期变化

五、体内免疫活化T细胞功能亚群变化

初始T细胞（Naïve T）经免疫活化后分化为不同功能亚群，分别维持效应功能和记忆功能。我们的研究结果显示，AA小鼠脾脏中供者T细胞经免疫活化后CD4^+^与CD8^+^细胞中Naïve T细胞比例均迅速下降，并由前期的中枢记忆T细胞（T_CM_）为主转变为效应记忆T细胞（T_EM_）为主，T_EM_比例在移植5天后维持在80％以上（图5A）。与脾脏内T细胞不同的是，骨髓内CD4^+^细胞中效应T细胞（T_E_）比例维持低水平，而CD8^+^细胞中T_E_比例高于CD4^+^细胞，且在第7、9天有上升趋势，提示CD8^+^细胞在AA模型骨髓造血损伤中发挥更重要的直接效应功能（图5B）。

**图5:**
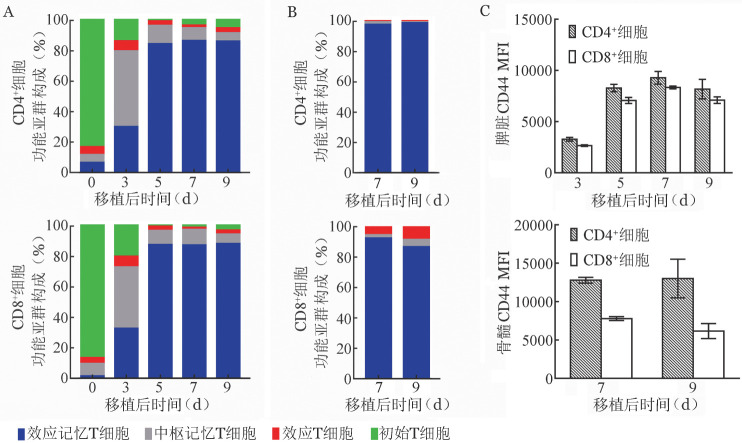
再生障碍性贫血（AA）小鼠体内免疫活化T细胞功能亚群变化 A：AA小鼠脾脏内供者来源T细胞功能亚群比例；B：AA小鼠骨髓内供者来源T细胞功能亚群比例；C：AA小鼠脾脏与骨髓内供者来源T细胞CD44平均荧光强度（MFI）

此外，对CD44分子表达水平的分析显示移植后脾脏内CD4^+^与CD8^+^细胞中CD44 MFI持续升高，至第7天开始下降，但第9天仍维持在较高水平，而T细胞进入骨髓后CD44 MFI呈下降趋势（图5C），该结果也提示移植后第5～9天为T细胞迁移至骨髓的关键时期。

## 讨论

免疫活化T细胞介导的AA小鼠是研究AA发病机制及探索新型治疗方法的有效模型。2004年Young团队首次通过照射联合淋巴结细胞输注的方式建立该小鼠模型[6]，其后也有研究通过照射联合脾脏细胞输注建立AA小鼠模型[7]–[8]，小鼠骨髓内广泛T细胞浸润与IFN-γ、TNF-α等负造血调控因子的分泌均提示两种方式建立的模型中起关键作用的为T细胞，而非B细胞。并且，与单纯大剂量淋巴结细胞输注建立的免疫诱导骨髓损伤的模型相比，照射联合小剂量淋巴结细胞输注的方式更不易引起移植物抗宿主病相关的皮肤、肝脏等组织损伤[9]。同时，研究表明经异基因活化的T细胞还可以免疫损伤同基因造血干细胞[6]，因此该模型可以很好模拟临床重型AA的发生。但由于小鼠缺乏临床上输血、营养支持的支持治疗，此模型小鼠生存时间仅2～3周。因此，掌握该模型中T细胞功能的动态变化在研究发病机制及探索新型治疗方法，尤其是免疫抑制治疗中显得格外重要。

既往研究显示，AA小鼠的血小板与骨髓有核细胞数目在输注后第7天开始低于单纯照射组小鼠[10]，表明输注后第7天受体小鼠骨髓开始经受明显的免疫打击，这与供者T细胞在骨髓内的浸润时间点相吻合，即输注后第3～7天供者T细胞缓慢进入骨髓，第7天以后开始大量扩增，我们的研究也验证了该结果。此外，本研究我们发现，输注第5天后受体小鼠脾脏内供者T细胞数目开始逐渐减少，而骨髓内T细胞数目逐渐增多，经过对供者T细胞的细胞周期分析发现，脾脏内供者T细胞中S/G_2_/M期细胞比例在移植后第5天开始下降，同期骨髓内T细胞中S/G_2_/M期细胞比例高于脾脏，表明骨髓中第7天以后大量浸润的T细胞部分来源于脾脏，部分为T细胞进入骨髓内再次经历增殖高峰所致。对比CD4^+^与CD8^+^细胞的细胞周期，从输注后第3天开始，脾脏与骨髓内处于S/G_2_/M期的CD8^+^细胞比例均持续高于CD4^+^细胞，这也从细胞增殖水平解释了输注后CD4^+^/CD8^+^细胞比值持续下降至比例倒置的现象，与既往研究中骨髓内浸润的CD8^+^细胞远多于CD4^+^细胞[11]的结果相一致，而此结果可能由于早期脾脏内CD8^+^细胞较CD4^+^细胞活化更早、活化程度更高所致。

Naïve T细胞经抗原刺激活化后可分别分化为效应T细胞与记忆T细胞，根据我们的研究结果推断，AA小鼠模型中供者T细胞经异体活化后分化过程为：Naïve T→T_CM_→T_EM_→T_E_几个阶段，而非我们常规认识的Naïve T→T_E_→T_EM_/T_CM_的分化过程，这可能与AA模型中T细胞移植后的特性有关，即经静脉移植后的T细胞需经淋巴细胞再循环最终到达靶器官。在此过程中，Naïve T细胞活化后分化的T_CM_细胞，随后逐渐丧失淋巴结归巢受体CD62L的表达，迁移与活化相关分子CD44的表达得以维持，分化为T_EM_细胞，获得部分效应功能，最终到达靶器官骨髓后部分细胞丧失CD44的表达，分化为效应功能更强的T_E_细胞（此现象在CD8^+^细胞中更加突出），而未分化的T_EM_细胞同时维持增殖与效应功能，发挥持续的骨髓损伤作用[12]–[13]。并且我们的研究结果表明由Naïve T细胞分化为T_EM_细胞的过程迅速，在输注后5天内即可完全转变，这可能也是部分免疫抑制治疗延迟至第5天开始时并不能阻止AA小鼠骨髓衰竭发生的原因。

综上，AA小鼠模型中供者T细胞进入受体小鼠后功能状态变化迅速，输注后第3～5天为其功能状态转变的关键时期，第5～9天是其进入骨髓的主要节点，并且其中CD8^+^细胞发挥主要的造血免疫损伤作用，因此选择合适的治疗时机与检查时间点及靶向CD8^+^细胞可以更好利用此小鼠模型探索AA的发病机制并寻求新的治疗手段。
